# Functional chitosan gel coating enhances antimicrobial properties and osteogenesis of titanium alloy under persistent chronic inflammation

**DOI:** 10.3389/fbioe.2023.1118487

**Published:** 2023-02-15

**Authors:** Ti Zhang, Xiaoyan Qin, Yuan Gao, Dan Kong, Yuheng Jiang, Xiang Cui, Miantong Guo, Junyu Chen, Feifan Chang, Ming Zhang, Jia Li, Pengbin Yin

**Affiliations:** ^1^ Department of Orthopedics, The Fourth Medical Center of Chinese PLA General Hospital, Beijing, China; ^2^ National Clinical Research Center for Orthopedics, Sports Medicine and Rehabilitation, Beijing, China; ^3^ College of Life Science and Technology, Beijing University of Chemical Technology, Beijing, China; ^4^ The First Medical Center of Chinese PLA General Hospital, Beijing, China; ^5^ Department of Orthopedics, General Hospital of Southern Theater Command of PLA, Guangzhou, China; ^6^ International Hospital, Peking University, Beijing, China

**Keywords:** titanium alloy, persistent chronic inflammatory environment, functional coating, anti-inflammatory, bacteriostasis, bone formation

## Abstract

Titanium is widely used as surgical bone implants due to its excellent mechanical properties, corrosion resistance, and good biocompatibility. However, due to chronic inflammation and bacterial infections caused by titanium implants, they are still at risk of failure in interfacial integration of bone implants, severely limiting their broad clinical application. In this work, chitosan gels crosslinked with glutaraldehyde were prepared and successfully loaded with silver nanoparticles (nAg) and catalase nanocapsules (n (CAT)) to achieve functionalized coating on the surface of titanium alloy steel plates. Under chronic inflammatory conditions, n (CAT) significantly reduced the expression of macrophage tumor necrosis factor (TNF-α), increased the expression of osteoblast alkaline phosphatase (ALP) and osteopontin (OPN), and enhanced osteogenesis. At the same time, nAg inhibited the growth of *S. aureus* and *E. coli*. This work provides a general approach to functional coating of titanium alloy implants and other scaffolding materials.

## Introduction

Titanium (Ti) is one of the most commonly used materials in orthopedic implants due to its good biocompatibility, excellent corrosion resistance, and outstanding mechanical properties (Xu et al., 2020; Wang et al., 2021). Although Ti alloys implants have achieved good efficacy in fixing fractures, correcting deformities, and joint replacements, there is still limitation of Ti alloy implants, especially early bacteria adhesion and then biofilm formation on the implant surface, leading to implant-associated infections, which is the main cause of the high rate of implant failure in clinical practice (Muruve et al., 2017; Kim et al., 2020). The persistent chronic inflammatory response that results in poor integration of the Ti implant interface with bone is one of the main causes of implant failure. When implanting an osseous biomaterial, the cues for the onset of the inflammatory response begin with preparation for surgery (Franz et al., 2011), and even aseptic inflammation associated with the nature of the implant ‘foreign body’ can lead to poor integration of the material with the bone (Chuang et al., 2002; Chrcanovic et al., 2014a; Chrcanovic et al., 2014b). Infection of the tissues surrounding the implant interface caused by bacterial infection also contributes to the development of a persistent chronic inflammatory response at the implant site, which can severely inhibit osseointegration and even lead to non-union (Gao et al., 2019; Wu et al., 2021).

The initial immune response triggered by the implantation of orthopedic biomaterials is mainly induced by innate immune cells of the myeloid lineage (Anderson, 1988; Donath et al., 1992), which secreted signaling molecules, such as chemokines and cytokines, create a microenvironment at the implantation site that directly determines the success or failure of the integration between the material and the bone. Macrophages, as the first innate immune cells to come into contact with the implant surface after implantation, are rapidly activated after migration to the injury site following tissue injury and present two functional phenotypes, one of which is a macrophage phenotype that promotes an inflammatory response. This macrophage phenotype is found to be disproportionately present in the clinic around pure titanium grafts (Rao et al., 2012; Mantovani et al., 2013). Such macrophage-mediated persistent chronic inflammation is detrimental to bone repair. The production of persistent tumor necrosis factor (TNF-α), interleukin 6 (IL-6), inducible nitrogen oxide synthase (iNOS), and reactive oxygen species (ROS) such as H_2_O_2_ can severely alter osteoblast activity and differentiation capacity leading to bone resorption (Devasconcellos et al., 2012; Muruve et al., 2017; Li et al., 2020). Therefore, how to effectively improve osteogenesis and integration of the bone-implant interface on the surface of the Ti alloy implant in a persistent chronic inflammatory environment remains the focus of bone implant research.

Modification of the surface of bone implants has become an effective strategy for promoting bone formation and enhancing the integration of bone graft interfaces. Zhu et al. (2015) used inductively coupled plasma-based dry etching techniques, bone formation is promoted by altering the microstructure of the surface of the material. Gulati et al. (2017), on the other hand, significantly improved stem cell differentiation through the coexistence of micron-scale spherical particles and nanoscale vertical tubular arrays of microsurface structures constructed by 3D printing and anodic oxidation techniques. At the same time, Zhao et al. (2020) conferred excellent antibacterial capacity and osseointegration capacity on steel plates by preparing a functional coating of a zinc oxide/collagen type I composite (Col-I). It was also found that coating titanium alloy surfaces with metallic tantalum successfully activated the Wnt/beta signaling pathway and transformed the growth factor beta/fruit fly parent against decaying proteins (Smad), thus accelerating natural mineral deposition in body fluids and effectively promoting osteogenesis. However, these approaches have focused on the effects of biomaterials on BMSCs and osteoblasts.

In contrast, integrating the implant-bone interface is a complex biological process with immune-inflammatory responses triggered by various cell types. Therefore, it is essential to focus on the regulatory effect of titanium implant surface modification on osteoblasts in the microenvironment of persistent inflammation after implant implantation. In our pre-experiments, our team demonstrated that catalase effectively breaks down H_2_O_2_ in inflammatory environments and reduces the expression of associated pro-inflammatory cytokines to effectively treat inflammatory related diseases (Qin et al., 2020; Li et al., 2021; Yan et al., 2022). And our group developed nanocapsules based on the formation of free radical reactions to provide reasonable protection for further applications of catalase in complex environments *in vivo* (Zhu et al., 2015; Tian et al., 2016; Zhang et al., 2017; Xu et al., 2019).

**SCHEME 1:**
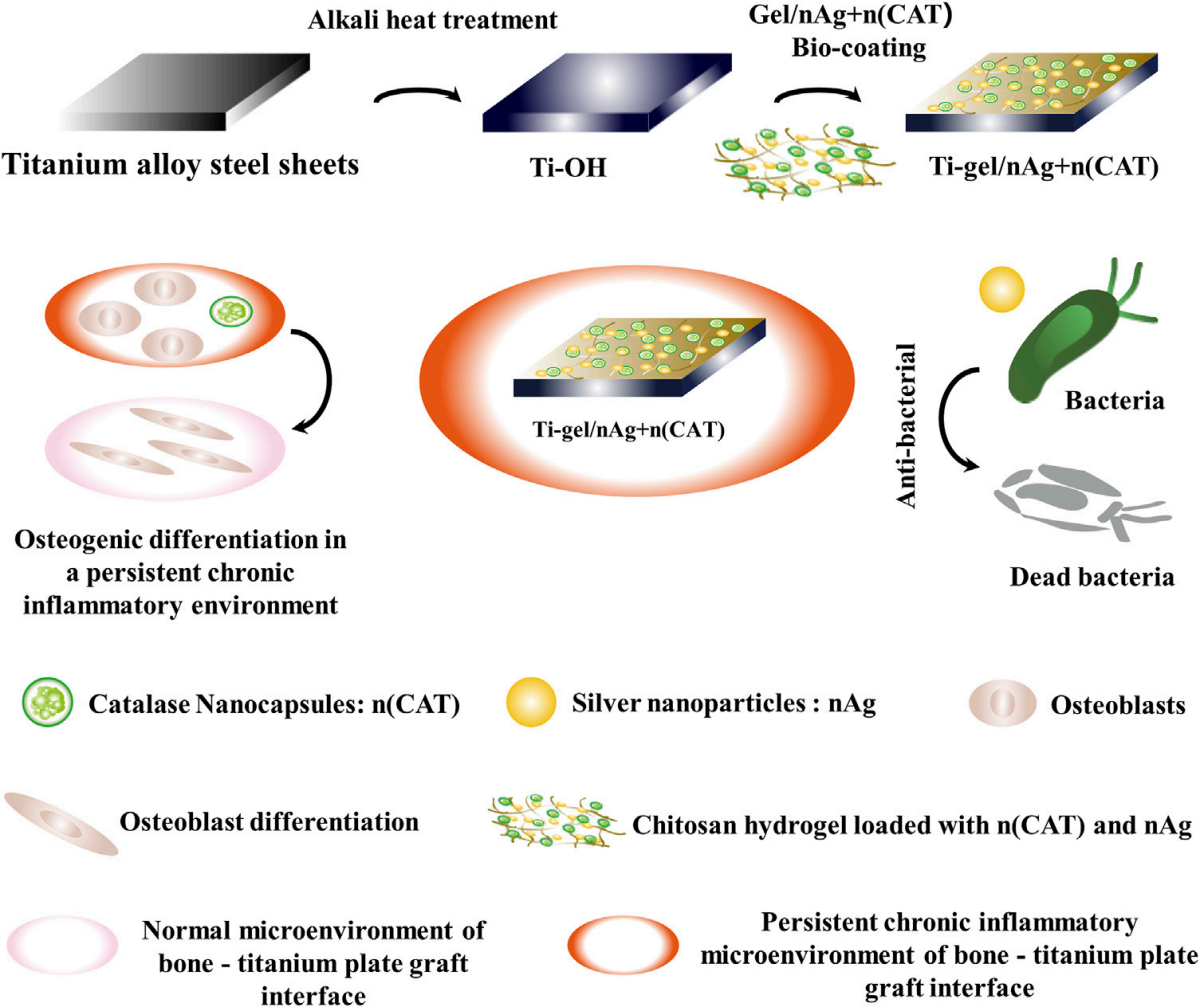
Schematic approach to antimicrobial and osteogenic differentiation in a persistent chronic inflammatory environment after coating titanium alloy steel plates with chitosan gel loaded with catalase nanocapsules (n (CAT)) and silver nanoparticles (nAg). The nano-silver in the gel coating provides effective antibacterial protection. At the same time, catalase nanocapsules effectively reduce the inhibition of osteoblast differentiation by removing hydrogen peroxide from the persistent chronic inflammatory environment and ultimately promote osteoblast differentiation by improving the persistent chronic inflammatory environment at the bone-titanium alloy graft interface.

In this work, we prepared chitosan gel crosslinked with glutaraldehyde to coat the titanium alloy surface, in which silver nanoparticles (nAg) and catalase nanocapsules (n (CAT)) were loaded. Based on the experimental results, the presence of nAg conferred an excellent antibacterial ability on the titanium alloy (Chimutengwende-Gordon et al., 2014; Huang et al., 2014; Cochis et al., 2015; Unosson et al., 2015; Cheng et al., 2022; Xiong et al., 2022; Yazdani-Ahmadabadi et al., 2022). Furthermore, osteoblast differentiation experiments found that n (CAT) effectively relieved inhibition of osteoblast differentiation in a chronic inflammatory environment and promoted osteogenesis; this could ultimately help improve the immunoinflammatory microenvironment at the implant-bone interface, providing a new idea for better integration of the implant-bone interface (Scheme 1).

## Materials and methods

### Materials

All reagents were used as received unless otherwise specified. Sandpaper was purchased from 3M (Minnesota, America). Silver nanoparticles were purchased from Feynman Nanomaterials Technology Co. Ltd (Liaoning China). Chitosan was obtained from Source leaf (Shanghai, China). Glutaraldehyde, acetic acid, nitric acid, hydrofluoric acid, sodium hydroxide, and sodium bicarbonate were purchased from Macklin (Shanghai, China). *Staphylococcus aureus* (*S. aureus,* CMCC26003) was obtained from Haibo Bio (Qingdao, China). *Escherichia coli* (*E. coli*, ATCC25922) was purchased from Xuanya Biotechnology Co. Ltd (Shanghai, China). Catalase (CAT) from Aspergillus niger was purchased from Sunson Industry Group (Beijing, China). Trypsin was purchased from Sigma Aldrich (St. Louis, MO), 2-methacryloyloxyethyl phosphorylcholine (MPC) was obtained from Anqing Kuangyou Biomaterials Co. Ltd. N-(3-Aminopropyl) methacrylamide hydrochloride (APM) was purchased from HEOWNS (Tianjin, China). N,N-methylenebisacrylamide (BIS), and ammonium persulfate (APS) were obtained from FuChen (Tianjin, China). N,N,N′,N′-tetramethylethylenediamine (TEMED) was purchased from Sangon Biotech (Shanghai, China). Mouse peritoneal macrophages (RAW264.7) were obtained from Northana Bio (Qingdao, China). Nutritional agar, Cell Counting Kit-8 (CCK-8), Bicinchoninic Acid (BCA) Protein Assay Kit, Catalase Activity Assay Kit, Endotoxin Erasol, phalloidin and DAPI were purchased from Beijing Solarbio Science and Technology Co. Ltd. (Beijing, China). Mouse TNF-α enzyme-linked immunosorbent assay (ELISA) kit, mouse IL-6 ELISA kit, and mouse IL-10 ELISA kit were purchased from PeproTech (Suzhou, China). BCA protein concentration determination kit, fluorescein isothiocyanate (FITC), dexamethasone, ascorbic acid, sodium β-glycerophosphate, Alizarin Red Staining Solution, 4% paraformaldehyde solution, and 25% glutaraldehyde solution were also obtained from Solarbio (Beijing, China). DMEM, α-MEM medium, penicillin/streptomycin (P/S, 1%), and fetal bovine serum (FBS) were purchased from Gibico (Australia). BCIP/NBT alkaline phosphatase (ALP) color development kit, Live/dead cell double staining kit (Calcein AM/PI), and CCK-8 kit were purchased from Biyuntian (Shanghai, China). Osteopontin (OPN) was obtained from Abcam (United Kingdom). Ultrapure water with a resistivity of 18.2 MΩ cm was used throughout.

### Instruments

Transmission electron microscopy (TEM) images were acquired on the Tecnai T12 cryo-electron microscope (FEI) using an acceleration voltage of 120 kV. Dynamic light scattering (DLS) measurements were performed on a Zetasizer Nano instrument (Malvern) with a 10-mW helium-neon laser and thermoelectric temperature controller. FTIR measurements were performed using an FTIR spectrometer (Nicolet 6700, Thermo). Fluorescence intensity imaging was performed using an automatic multifunction imaging analysis system (OI600-MF-Touch). The rheological properties of chitosan hydrogels were measured at 37°C using an ARES-LS2 rheometer (TA Instruments, New Castle, DE). Cell adhesion to the surface of the material, the cytoskeleton, and the fluorescence images of the OPN were observed using a confocal laser scanning microscope (Leica TCS SP8). Fluorescence intensity was measured with a microplate reader (Multiskan GO, Thermo). Fluorescently stained cells were imaged with an upright fluorescence microscope (Nikon, Japan). UV absorption measurement was used by NANODROP (Thermo).

### Synthesis

**Synthesis of Ti-OH.** First, the surface of the titanium alloy plate was sanded with 800, 1200, and 2000 grit sandpaper until the surface was smooth and flat, and the polished titanium alloy plate was named Ti alloy plate. The Ti alloy plate was then immersed in a mixed acid solution of nitric acid (25%, v/v), hydrofluoric acid (3%, v/v), and water for 1 min and then removed and rinsed with deionized water. After drying the Ti surface with dust-free paper, it was placed in a 2 mol/L sodium hydroxide solution and reacted at 80°C for 4 h to obtain treated Ti treated with alkali (Ti-OH). Soak Ti and Ti-OH in a 75% ethanol solution for 15 min to obtain sterile Ti and Ti-OH.

**Synthesis of Chitosan Gels.** 7.5 mg of chitosan was dissolved in 185 μL of acetic acid solution (2%, v/v), and 15 μL of glutaraldehyde solution (2%, v/v) was added to the completely dissolved chitosan solution, mixed well and left to form a gel at room temperature for 2 h. To obtain sterile chitosan gels, the chitosan was pretreated with UV light for 30 min and the acetate and glutaraldehyde solutions were filtered through a 0.22 μm filter membrane on an ultra-clean bench. The gels were prepared on the ultra-clean table described above to obtain sterile gels.

**Synthesis of n(CAT).** CAT nanocapsules, denoted as n (CAT). It was synthesized as previously reported (Qin et al., 2020). Briefly, CAT was thoroughly mixed with MPC, APM, BIS, TEMED, and APS in a molar ratio of 1:21600: 2400: 2400: 4800: 2400 in 10 mM pH 7.0 PBS buffer. A free radical polymerization reaction was initiated by the final addition of APS and TEMED. The reaction was carried out at 4°C for 2 h and then dialyzed in 10 mM pH 7.0 PBS buffer using a cellulose membrane (MWCO 100 kDa) to remove unreacted monomers and initiators. The dialyzed solution is then purified by passing it through a phenyl sepharose CL-4B column (GE) in the presence of a 10xPBS elution buffer. Finally, the purified solution was concentrated using an ultrafiltration tube to obtain n (CAT). Sterile n (CAT) was obtained by filtration using a 0.22 μm membrane on an Ultraclean table.

**Synthesis of n(CAT)-FITC.** For imaging purposes, n (CAT) was fluorescently labeled with FITC (Ex 490 nm, Em 525 nm). Briefly, n (CAT) and FITC were mixed in a 1: 5 M ratio and incubated at room temperature for 48 h in the dark. Then, the combination used to be considerably dialyzed into 10 mM pH 7.0 PBS buffer with the use of a cellulose membrane (MWCO 10 kDa) to remove unreacted FITC and eventually obtain FITC-labeled n (CAT) (denoted as n (CAT)-FITC).

**Synthesis of Gel/nAg, Gel/n(CAT) and Gel/nAg + n(CAT).** Chitosan gels loaded with nAg, n (CAT) and nAg + n (CAT) were denoted as gel/nAg, gel/n (CAT) and gel/nAg + n (CAT). According to the steps above in the preparation of chitosan, nAg or n (CAT) was added to the completely dissolved chitosan solution, where the concentration of nAg was 20 ppm and the enzyme activity of n (CAT) was added at 16 μg/ml, well mixed. The 2% glutaraldehyde solution was then added and well mixed to obtain the drug-loaded pregel solution, and the drug-loaded pregel solution was left to stand at room temperature for 2 h to obtain gel/nAg, gel/n (CAT) and gel/nAg + n (CAT). After the sterile raw material for the preparation of drug-loaded chitosan gels was obtained according to the method described above, the gels were prepared on an ultraclean table to obtain sterile gel/nAg, gel/n (CAT), and gel/nAg + n (CAT).

**Synthesis of Ti-gel, Ti-gel/n(CAT) and Ti-gel/nAg.** The chitosan gels and drug-loaded chitosan gel-coated titanium alloy steel sheets were denoted as Ti-gel, Ti-gel/n (CAT), Ti-gel/nAg, Ti-gel/nAg + n (CAT) and Ti-gel/nAg + n (CAT)-FITC. On the basis of the above methods, sterile Ti-OH, chitosan gel, and drug-carrying chitosan gel were prepared. Briefly, after adding 200 μL drops of pre-gelatinization solution to the Ti-OH surface and leaving it for 3 min to completely gel, it was then placed in a sterile well plate and placed in an oven at 37°C overnight resulting in sterile Ti-gel, Ti-gel/n (CAT), Ti-gel/nAg, Ti-gel/nAg + n (CAT), and Ti-gel/nAg + n (CAT)-FITC. The above operation was performed in a sterile ultra-clean table.

### Cell culture

**Isolation and Culture of BMSCs.** Bone marrow-derived MSCs (BMSCs) were collected from the femur and tibia of 1-month-old New Zealand rabbits. Approximately 2–5 ml of fresh bone marrow was removed from the rabbit femurs and tibias and cultured in α-MEM (10% fetal bovine serum, 1% P/S) for 5 days (37°C, 5% CO_2_, 95% relative humidity). When non-adherent cells were removed, most adherent cells were considered BMSCs. After reaching 80%–90% fusion, primary BMSCs were passaged in a 1: 3 ratio with 0.25% trypsin/EDTA. Subsequently, 2-4 generations of BMSCs were used for *in vitro* cytotoxicity and value-added adhesion assays.

**Isolation and Culture of Osteoblasts.** Osteoblasts were collected from the skulls of newborn C57 mice. The skulls were digested in 500 μl of 0.25% trypsin solution at 37°C for 10 min, followed by washing with a-MEM medium. The digestion reaction was then continued by adding 600 μl of 0.2% (2 mg/ml) type II collagenase solution for 30 min at 37°C. The skulls were further excised and placed in a type II collagenase solution for 60 min at 37°C. The digest was centrifuged at 1500 r/min for 5 min, the supernatant was discarded and the precipitated fraction was resuspended in α-MEM containing 10% FBS and 1% P/S. Resuspension was transferred to culture flasks (37°C, 5% CO_2_, 95% relative humidity), and the culture medium was changed every 2–3 days (Zheng et al., 2020). The primary osteoblasts obtained were used for subsequent osteogenic differentiation experiments.

**Culture of Mouse Peritoneal Macrophages (RAW264.7).** Mouse peritoneal macrophages (RAW264.7) were purchased from Northana Bio and cultured using a DMEM medium containing 10% FBS and 1% P/S for subsequent cytokine assay experiments.

### Determination of the concentration and activity of CAT and n (CAT)

**Protein Concentration Assay.** The concentration of CAT and n (CAT) was determined by optical absorption measurements using an extinction coefficient of ε = 324000 M^−1^ cm^−1^ at 405 nm (Samejima and Yang, 1963).

**Catalase Activity Assay**. The activity of CAT and n (CAT) was tested using a catalase activity assay kit. Briefly, 1 ml of H_2_O_2_ solution (pH = 7.4, 0.1 M HEPES buffer, H_2_O_2_ concentration 0.03% w/v) and 35 µL of the sample were added to a 1 ml quartz cuvette. After mixing for 5 s, the absorbance at 240 nm was measured immediately (A1) and after 1 min (A2). The following equation calculates the activity of catalase:

$$Catalase\;activity\;\left(U/mL\right)=\left[∆A\times V_{total}÷\left(\varepsilon \times d\right)\;10^{6}\right]÷V_{sample}÷T=678\times ∆A$$

Where ∆A=A1−A2 ; $V_{total}$ stands for the total volume of the reaction system, 1.035 Ml; ε stands for the molar absorptivity of H2O2, 43.6 L/mol/cm; d stands for the optical path of the cuvette, 1cm; $V_{sample}$ stands for the volume of the sample added, 0.035 ml; T stands for the reaction time, 1min.

### Characterizations of the n (CAT)

**Dynamic Light Scattering (DLS) Measurement.** The particle size distribution of CAT and n (CAT) was measured using a Zetasizer Nano instrument. The instrument is equipped with a 10-mW HeNe laser and a thermoelectric temperature controller to measure the particle size of CAT and n (CAT) at a measurement temperature of 25°C and a scattering angle of 90° (n = 3).

**Transmission Electron Microscopy (TEM)**. The sample was prepared as previously described (Tian et al., 2016; Qin et al., 2020). Briefly, a 1% (w/v) solution of phosphotungstic acid was used to stain n (CAT) and was added dropwise to the copper web. Finally, the morphology of n (CAT) on the copper web was observed using an HT7700 field emission electron microscope at 100 kV.

### Characterizations of chitosan gels

**Rheology (TA) Measurements of Chitosan Gels**. The mechanical properties of the chitosan hydrogels were determined using an ARES-LS2 rheometer (TA Instruments, New Castle, DE). The rheometer has a 25 mm stainless steel upper cone and a temperature-controlled Peltier base plate on which a 2 cm diameter, 2 mm thick hydrogel sample was placed. The samples were dynamically scanned at 37°C, 1Hz, and with a 1% strain applied for 900 s to determine the mechanical properties of the hydrogel (n = 3).

**Fourier Transform Infrared Spectroscopy (FT-IR).** Infrared spectroscopy was performed on freeze-dried chitosan hydrogel samples using a Jasco FT/IR-6200 spectrometer (Jasco, OK, United States) equipped with a deuterated triglyceride sulfate detector (Pike Technologies, WI, United States). Chitosan hydrogels were prepared as previously described and then freeze-dried at -20°C before subjecting them to FTIR analysis. Thirty-two scans from 600 to 4000 cm ^−1^ were taken for each measurement to obtain spectra with a nominal resolution of 4 cm ^−1^.

### Characterizations of the Ti, Ti-OH, and Ti-gel/nAg + n (CAT)

**SEM-EDS**. The morphology and element composition of the surface of Ti, Ti-OH, Ti-gel/nAg + n (CAT) samples were measured using a field-induced emission scanning electron microscope (FE-SEM; Sigma500, Zeiss, Germany) equipped with an energy dispersion X-ray spectrometer. The entire observation process was operated at a constant accelerating voltage of 5 kV in a vacuum environment. Before imaging, samples were sputter coated with gold to increase conductivity.

**Fluorescence Imaging.** Optical imaging was used to further confirm the successful coating of fluorescently labeled n (CAT) on Ti surfaces achieved using an automated multifunctional imaging analysis system (OI-600MF-Touch, Guang Yi Bio, Guangzhou).

**Water Contact Angle (WAC) Assay** Hydrophilic testing of Ti, Ti-OH and Ti-gel/nAg + n (CAT) surface using a contact angle goniometer (DSA100, Dataphysics Instruments GmbH, Filderstadt, Germany). Briefly, the material was placed on the stage and raised so that the material being examined would come into contact with water droplets from the injector. Then, the stage was lowered to remove the droplets. Finally, a photo of the water droplets was taken after 10 s. The five-point fitting becoming approach was once used to measure the static water contact angle.

### Antibacterial assay

**Bacterial Culture.** Nutrient agar was dissolved in deionized water at 10% (m/v) to obtain a solid medium for bacteria; mix 10 g of peptone, 3 g of beef paste, and 5 g of sodium chloride dissolved in 1 L of distilled water to obtain a liquid medium. Both mediums were autoclaved at 121°C for 20 min and used to culture *E. coli* and *S. aureus.* Individual colonies were picked and placed in 5 ml of liquid medium and incubated in a constant temperature incubator at 37°C with shaking at 180r/min for 12 h to obtain freshly cultured bacterial broth.

**Inhibition Zone Assay.** The antimicrobial activity of Ti-Gel/nAg was studied by inhibition zone assay analysis. 1 ml of freshly cultured mycelium was taken and diluted in a 1000-fold gradient with 1xPBS buffer. Then 100 μL of the diluent was added to the solid medium. After uniform coating, the material was placed in the middle of a solid medium and incubated for a total of 12 h at 37°C to observe the formation of the inhibition zone.

**Bacterial Survival Rate Assay**. After centrifugation with 1 ml of fresh bacterial solution, the supernatant was discarded, resuspended in a sterile 1 ml of 1× PBS buffer, further diluted 1000 times gradiently, then the material was immersed in a diluted 4 ml bacterial solution and co-incubated at 37°C for 12 h. After co-culture, the co-cultivated bacterial solution was again diluted in a 1000-fold gradient using 1xPBS buffer. After incubation at 37°C for 24 h, 100 uL of diluted bacterial solution was taken and evenly spread on solid medium and colonies on the surface of agar plates were counted and the experiment was repeated 3 times for each group (n = 3). The bacterial survival rate was obtained through the following equation.

$$Bacterial\;survival\;\left(\%\right)=\frac{Is}{Ic}\times 100\%$$

Where *Is* the number of colonies in the test sample and *Ic* is the total number of colonies of the control bacteria.

### Cytotoxicity of n (CAT) and nAg

Fourth-generation BMSCs were used to study the toxic effects of n (CAT) and nAg at the cellular level. First, BMSCs were seeded at a density of 5 × 10^3^ cells/well in 96-well plates and incubated overnight at 37°C to adhere them to the wall. Subsequently, 100 μL of the n (CAT) solution (100, 200, 500, 1000, and 2000 ng/ml) and the nAg solution (5, 10, 15, 20, 25 ppm), prediluted with serum-free medium, were added and incubated with cells for 24 h at 37°C. Finally, 10 μL of CCK-8 reagent was added to each well and incubated for 2–3 h at 37°C. The absorbance was measured at 450 nm using a multifunctional enzyme marker (EnSpire PerkinElmer) microplate reader. The absorbance value in the blank wells was 100%, which was used to convert the relative cell viability of the experimental group. (n = 5).

### Adhesion and proliferation assay of BMSCs on the surface of Ti and Ti-gel/nAg + n (CAT)

Fourth-generation BMSCs were used to study the ability of cells to adhere to Ti and Ti-gel/nAg + n (CAT) surfaces for value-added. Sterile Ti and Ti-gel/nAg + n (CAT) were prepared as previously described. Ti and Ti-gel/nAg + n (CAT) were pre-equilibrated in medium for 2 h. BMSCs were seeded on Ti and Ti-gel/nAg + n (CAT) surfaces at a density of 2.5 × 10^4^ cells per well. The surface of the material was rinsed 3 times with PBS to remove loosely attached cells, followed by adding 0.5 ml of α-MEM medium containing 10% CCK-8 and incubation at 37°C for 2–3 h before measuring the OD at 450 nm. The previous measurements were repeated on days 2, 3, and 4. At the same time, BMSCs were inoculated on the surface of Ti and Ti-gel/nAg + n (CAT) materials in the same way; cells were stained with Ghost Cyclin/DAPI labeled with Rhodamine and Calcein AM/PI at different time points, then live-dead and cell morphology were observed using fluorescence microscopy (Nikon japonica) and confocal fluorescence microscopy (Leica TCS SP8), respectively.

### Osteogenic differentiation assay of osteoblasts cultured on the surface of gel and Gel/n (CAT) in a persistent chronic inflammatory environment

Osteoblasts were used in a persistent chronic inflammatory environment to study the ability of osteogenic differentiation on different surfaces of materials. The sterile gel and gel/n (CAT) were prepared using the method described above. First, by interfering with the formation of a persistent chronic inflammatory environment by adding 100 μM H_2_O_2_ to the osteoblast induction medium and then investigating the osteoblast differentiation capacity on the gel and gel/n (CAT) surface. Gel and Gel/n (CAT) were pre-equilibrated in medium for 2 h. Osteoblasts were inoculated on the Gel and Gel/n (CAT) surface at a density of 1 × 10^4^ cells per well. After 2 days of incubation, the α-MEM medium was replaced with an osteogenic induction medium and the culture was changed daily until days 7 and 14.

**Alizarin Red and Alkaline Phosphatase Staining.** Alizarin red staining was performed on day 7 of the osteogenic differentiation culture according to the previous method. Cells were fixed in 4% tissue fixative for 15 min, rinsed three times with 1xPBS solution, followed by Alizarin Red staining solution for 30 min. On day 14, alkaline phosphatase staining (ALP) was performed and the ALP working solution was prepared according to the instructions for the ALP kit (Beyotime, C3206). Cells were fixed in 4% tissue fixative for 15 min. They were washed three times with 1 x PBS solution and subsequently stained with ALP working solution for 60 min. All staining results were observed with a biological scanning electron microscope (SU8010 Thermo) and quantitatively analyzed using ImagePro software.

### Cytokine expression ability of macrophages on the surface of Ti and Ti-gel/n (CAT) in a persistent chronic inflammatory environment

RAW264.7 macrophages were used to study cytokine expression on different surface surfaces of materials in a persistent chronic inflammatory environment. Sterile Ti, Ti-gel/n (CAT) and chronic inflammatory environments were obtained according to the method previously described to study the anti-inflammatory capacity of macrophages on their surfaces. The macrophages were inoculated at 2 × 10^4^ per well on the surface of Ti and Ti-gel/nAg + n (CAT), and after 24 h of incubation, the cell supernatant was aspirated. The concentrations of TNF-α, IL-6, and IL-10 in the supernatant were measured using ELISA kits (PeproTech, Suzhou, China).

### OPN immunofluorescence staining of osteoblasts on Ti and Ti-gel/n (CAT) in a persistent chronic inflammatory environment *via* n (CAT)

Osteoblasts were inoculated onto the Ti and Ti-gel/n (CAT) surfaces at a density of 1 × 10^4^ cells per well according to the previous method. After 2 days of incubation, the α-MEM medium was replaced with an osteogenic induction medium. The medium was changed daily until day 7, when immunofluorescence staining for OPN was performed. Briefly, cells were fixed in 4% tissue fixative for 15 min, rinsed three times with 1 x PBS solution, followed by further treatment of the samples with 0.3%–0.5% Triton X-100, immediately followed by the addition of 10% goat serum for 1 h at room temperature to block non-specific binding. After adding primary antibodies to the samples overnight at 4°C, secondary antibodies Alexa Fluor 647 goat anti-mouse IgG (Abcam) were added and incubated at room temperature for 2 h. The cytoskeleton and nuclei were then treated with rhodamine-labeled ghost pencil cyclic peptide and DAPI. Finally, the OPN fluorescence images of the surfaces of different materials were observed by a fluorescence confocal microscope. (Nikon).

### Statistical analysis

As indicated, all results are presented as the mean ± standard error of the mean (s.e.m.). Three statistical methods, including T-test, one-way ANOVA and two-way ANOVA were used in this article. All statistical analyses were performed with Prism Software (Prism 8.0.1).

## Results and Discussions

### Characterization of gel, n (CAT) and Ti-gel/nAg + n (CAT)

#### Characterization of gel

**FIGURE 1:**
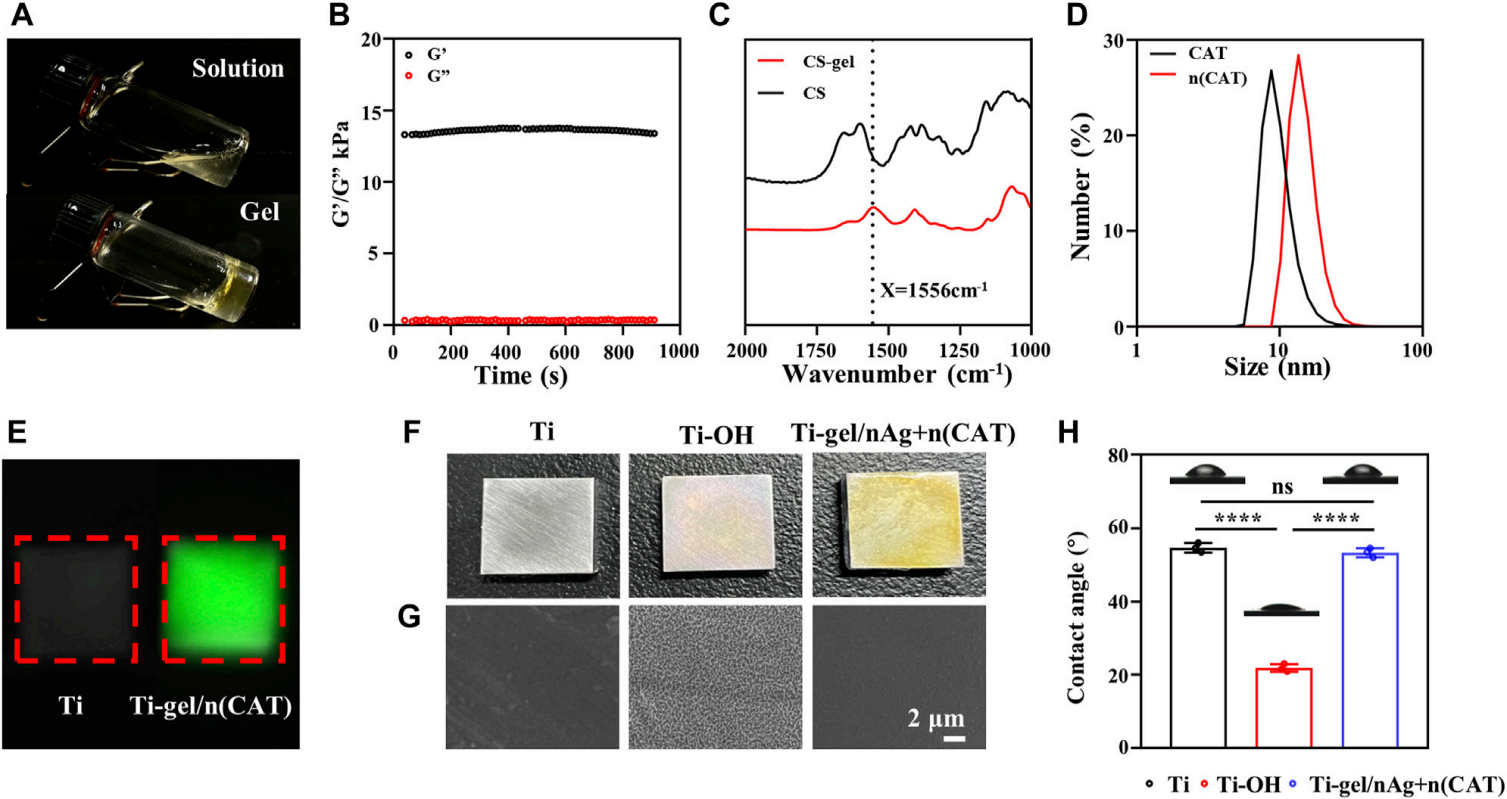
Characterization of gel, n (CAT), and Ti-gel/nAg + n (CAT). **(A)** Physical diagram of the gel. **(B)** Young’s modulus of the gel. **(C)** FT-IR spectra of chitosan and gel. **(D)** DSL of CAT and n (CAT). **(E)** Fluorescent images of Ti and Ti-gel/n (CAT). **(F and G)** Physical diagram and SEM of Ti, Ti-OH, and Ti-gel/nAg + n (CAT). **(H)** Ti, Ti-OH, and Ti-gel/nAg + n (CAT) water contact angle. (n = 3, ns means that there are no significant differences, ****p < 0.0001).

By preparing a chitosan hydrogel that can be loaded with bioactive substances, the surface of a titanium alloy steel plate is coated to impart bioactivity and its related characterization. Figure 1A shows the physical diagram of the chitosan solution in a gel. Its mechanical properties were measured using a rheometer. The results show that the elastic modulus of the chitosan gel was significantly higher than the viscous modulus (G'> G″), suggesting that the system was similar to a solid-like colloid and that the system was indeed transformed from a chitosan solution to a hydrogel colloid. The trend of the curve was stable, indicating that the chitosan gel cross-linked system was stable (Figure 1B). Compared to chitosan, the chitosan gel had a characteristic peak glutaraldehyde cross-linking band at 1556cm^−1^ (amide II). (Li et al., 2013). This shows that the chitosan hydrogel system is indeed formed by glutaraldehyde crosslinking (Figure 1C). These results are evidence of the successful synthesis of chitosan gels.

#### Characterization of n (CAT)

Catalase nanocapsules were prepared and characterized as previously described. The DLS results confirmed that the hydrodynamic radius of the CAT was 7.58 nm, while the hydrodynamic radius of n (CAT) reached 13.6 nm **(** Figure 1D). Further TEM results showed that n (CAT) has a spherical morphology with an average diameter of about 20 nm (Supplementary Figure S1A), which was consistent with the DLS results. This result indicated that the monomers and BIS cross-linkers incubated with CAT formed a thin layer of polymer shell around the CAT. These results collectively demonstrate the successful synthesis of n (CAT). Compared to CAT, n (CAT) exhibited a similar enzymatic activity (Supplementary Figure S1B), showing that the nanopolymer shell layer did not affect the CAT enzymatic activity, and the prepared n (CAT) can be used for subsequent experiments. Next, n (CAT) was fluorescently labeled with FITC to investigate the effect of loading n (CAT) and nAg for the gel formation of the chitosan solution. As shown in Supplementary Figure S1C, the chitosan solution loaded with n (CAT) and nAg formed a gel well and n (CAT) was evenly distributed in the gel. The gel/n (CAT)-FITC was further coated on the surface of Ti-OH, as can be seen by fluorescence imaging that n (CAT) was uniformly distributed on the surface of Ti-OH, indicating that the loading of n (CAT) by Ti-OH was achieved successfully (Figure 1E).

#### Characterization of Ti-gel/nAg + n (CAT)

The microscopic morphological changes of Ti, Ti-OH, and Ti-gel/nAg + n (CAT) surfaces were investigated. As can be seen in the physical image in Figure 1F, the surface of Ti-OH shows a blue metallic luster compared to Ti, while the surface of Ti-gel/nAg + n (CAT) after the drug-loaded chitosan gel coating is pale yellow. From further SEM images (Figure 1G), which showed that the Ti surface is smooth and that the Ti-OH surface showed a porous mesh structure compared to Ti. Further treatment with the drug-loaded gel coating restored the smooth surface of Ti-gel/nAg + n (CAT). Meanwhile, the hydrophilicity of different materials’ surfaces was measured by detecting the contact angle of these surfaces with water. The angle of contact with water on the surface of Ti-OH (21.8°) was found to decrease significantly compared to that of Ti (54.7°) (Figure 1H). In comparison, the water contact angle of the Ti-gel/nAg + n (CAT) surface after the drug-loaded chitosan gel coating was restored to 53.3°, basically the same as that of the untreated Ti surface. All of these results indicated that the Ti-OH surface was successfully coated with the drug-loaded chitosan gel and that the coating treatment did not affect the excellent hydrophilicity of Ti itself.

### Characterization of the anti-bacterial ability of Ti-gel/nAg

**FIGURE 2:**
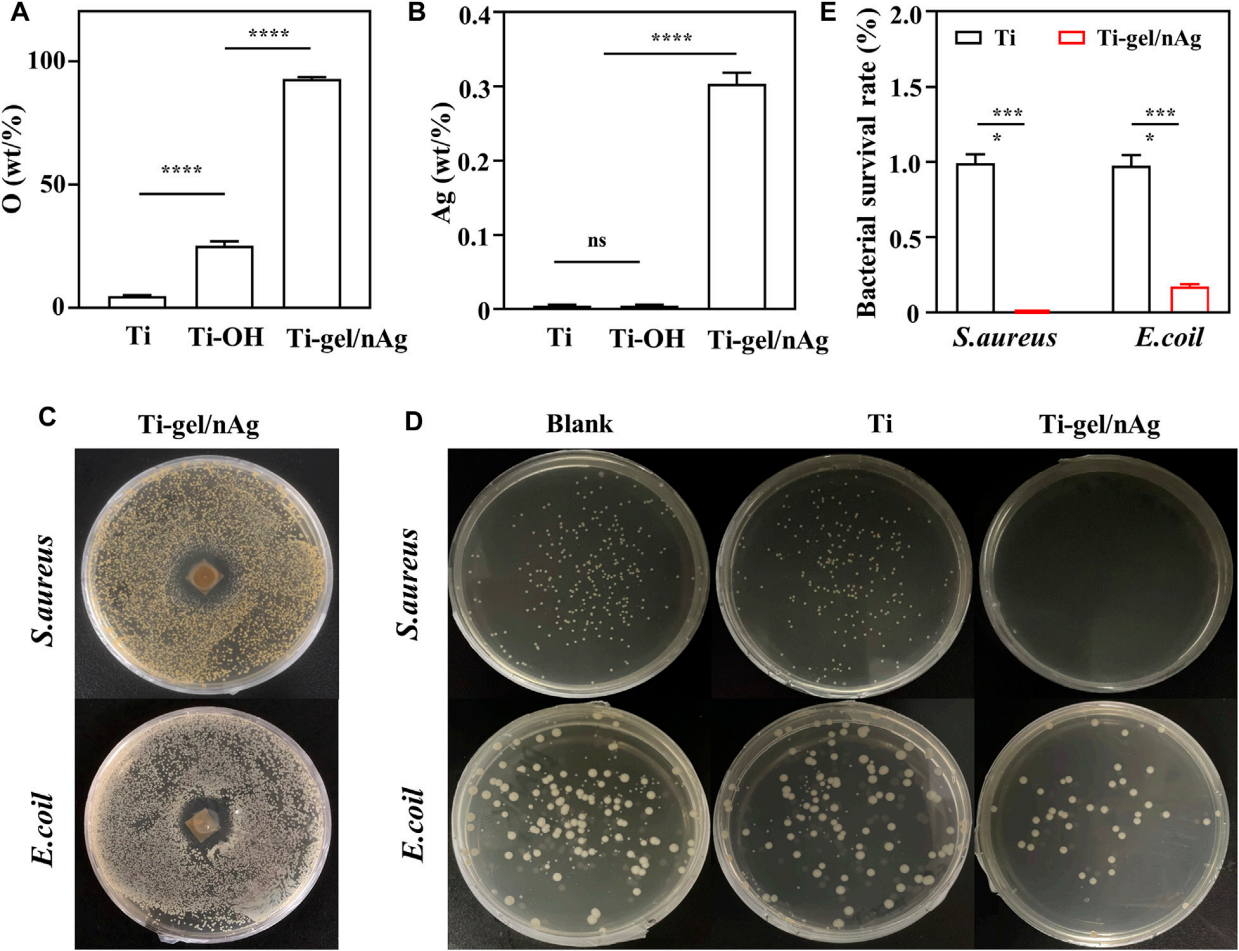
The surface elemental composition and antibacterial capacity of Ti and Ti-gel/nAg. **(A)** Relative elemental O and Ag **(B)** content of Ti, Ti-OH, and Ti-gel/nAg. **(C)** Inhibition zone of Ti and Ti-gel/nAg after co-culture with *S. aureus/E. coil* for 12 h **(D)** CFU of Ti and Ti-gel/nAg after co-culture with *S. aureus/E. coil* for 12 h **(E)** Bacterial survival of Ti and Ti-gel/nAg after co-culture with *S. aureus/E. coil* for 12 h (n = 3, ns means no significant difference, ***p < 0.001).

The Ti-OH surface was then coated with gel-nAg to investigate the antibacterial ability of Ti-gel/nAg. The elements on the Ti, Ti-OH, and Ti-gel/nAg surfaces were determined by EDS. Supplementary Figure S2A shows the full spectrum of the point sweep energy spectra of the Ti, Ti-OH, and Ti-gel/nAg surfaces. Further semiquantitative elemental analysis of O and Ag on their surfaces revealed a significant increase in O element on the Ti-gel/nAg surface compared to Ti (Figure 2A), which was due to the high amount of oxygen in chitosan. The Ag element content also showed the same variation as oxygen (Figure 2B), demonstrating a successful loading of nAg on the surface of titanium alloy steel plates. *S. aureus* and *E. coli* as representatives of Gram-positive and Gram-negative bacteria, respectively (Huang et al., 2019), these two bacteria were selected for inhibition zone and bacterial survival tests to investigate the anti-bacterial ability of Ti-gel/nAg. After co-culture of Ti-gel/nAg on agar plates coated with *S. aureus* and *E. coli*, a clear antibacterial circle appeared in the medium (Figures 2C, D), indicating that Ti-gel/nAg had some inhibitory ability on *S. aureus* and *E. coli* growth*.* Furthermore, as seen by the bacterial survival test (Figure 2E), in the control medium and Ti-treated, a similar number of colony units were observed to form, while, after Ti-gel/nAg treatment, no colony formation of *S. aureus* was observed in the medium. The same trend was observed in the results of the bacterial survival test for *E. coli*, with a significant reduction in the number of *E. coli* colonies in the medium after the Ti-gel/nAg treatment compared to the control and Ti-treated *E. coli* colonies. After calculating the bacterial survival rate, it was found that the survival rate of *S. aureus* in Ti-gel/nAg treatment was 0, and that of *E. coli* was only 0.17%. The above results demonstrate that Ti-gel/nAg has a good antibacterial ability.

### BMSCs adhesion and proliferation on Ti-gel/nAg + n (CAT)

**FIGURE 3:**
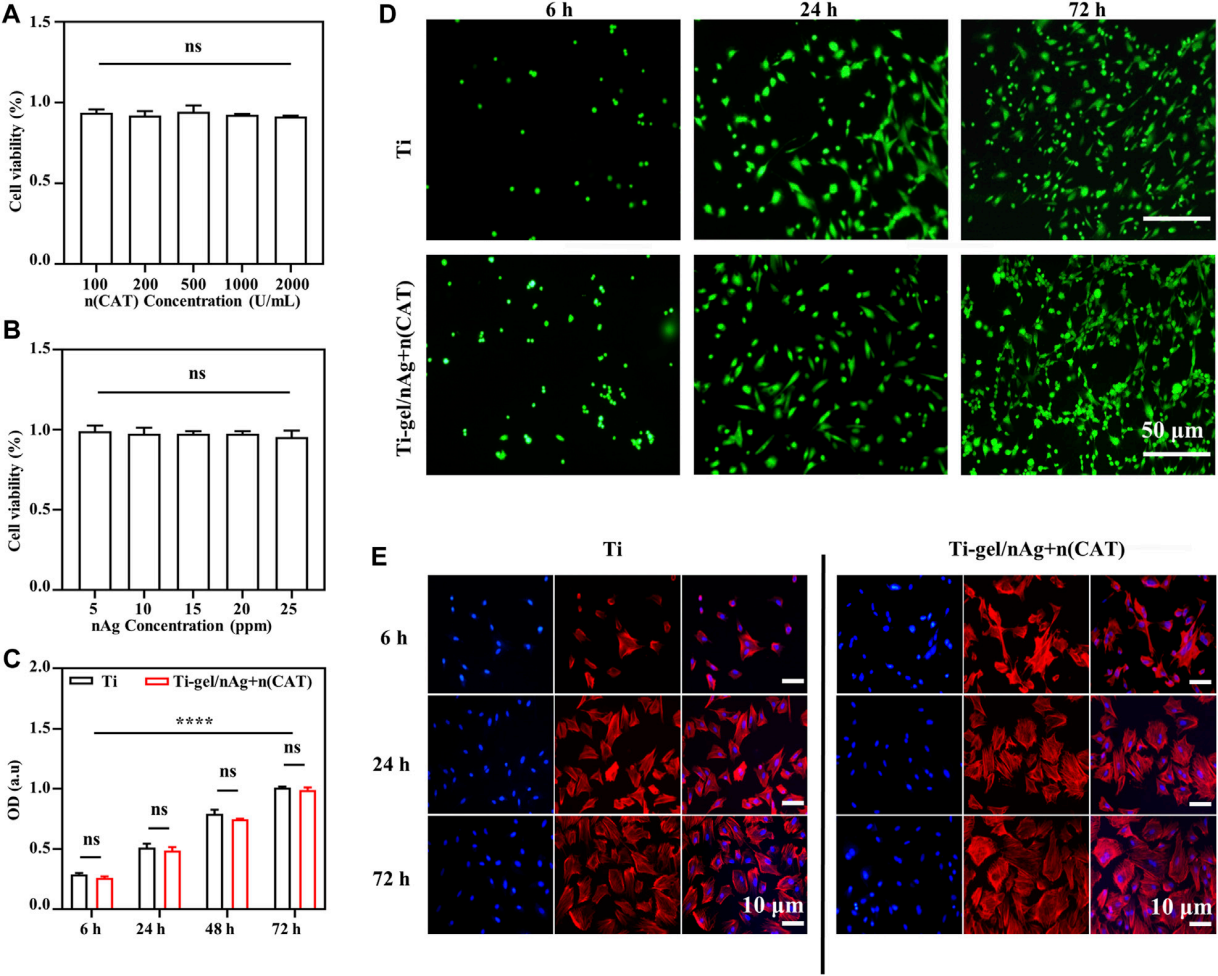
The adhesion and cell proliferation of BMSCs on the surface of Ti-gel/nAg + n (CAT). **(A,B)** Cell viability at different concentrations of n (CAT) and nAg. **(C,D)** BMSC fluorescence images adhered to different surfaces at different time points, and BMSC proliferation profiles were obtained by the CCK-8 assay. **(E)** Cell morphology of BMSCs growth on different surfaces at different time points. (n = 3, ns means that there are no significant differences, ****p < 0.0001).

To test the biocompatibility of Ti-gel/nAg + n (CAT) surfaces, BMSCs were selected to investigate the effects of drug-laden chitosan gel coating on the surface of titanium alloy steel plates on cell behavior, such as value-added adhesion and cell morphology. First, the cytotoxicity of n (CAT) and nAg was investigated, and it was found that the concentration of n (CAT) enzyme activity of 100-2000u/mL (Figure 3A) and the nAg concentration of 5–25ppm were not significantly toxic to cells (Figure 3B). Then, it was found that the OD values of BMSCs in Ti and Ti-gel/nAg + n (CAT) were not significantly different at different time points in adhesion and proliferation (Figure 3C), and the OD values gradually increased with time, indicating that the cells had a good proliferation ability on the material surface. Meanwhile, consistent results were also observed on the live-dead stained fluorescence images of the Ti and Ti-gel/nAg + n (CAT) surfaces (Figure 3D), and with good cell morphology on the Ti and Ti-gel/nAg + n (CAT) surfaces at all time points (Figure 3E). All of these results collectively demonstrate the good biocompatibility of Ti-gel/nAg + n (CAT) for further application *in vitro*.

### Effect of Gel/n (CAT) on osteoblasts osteogenic differentiation in a persistent chronic inflammatory environment *in Vitro*

ALP is a precise marker of the early stage of osteogenic differentiation. Meanwhile, calcium deposition is one of the vital signs and symptoms of late osteogenic differentiation, which can be specially examined using ARS. As a result of the opaque nature of the titanium alloy steel plate material, the staining phenomena ALP and ARS could not be observed. Gel/n (CAT) was used as a model for the application of Ti-gel/n (CAT) to investigate the effect of a persistent chronic inflammatory environment on osteogenic differentiation of osteoblasts on the surface of different materials. Osteoblasts on the surface of different materials were stained for ALP and ARS on day 7 and day 14 of osteogenic differentiation culture in a chronic inflammatory environment.

**FIGURE 4:**
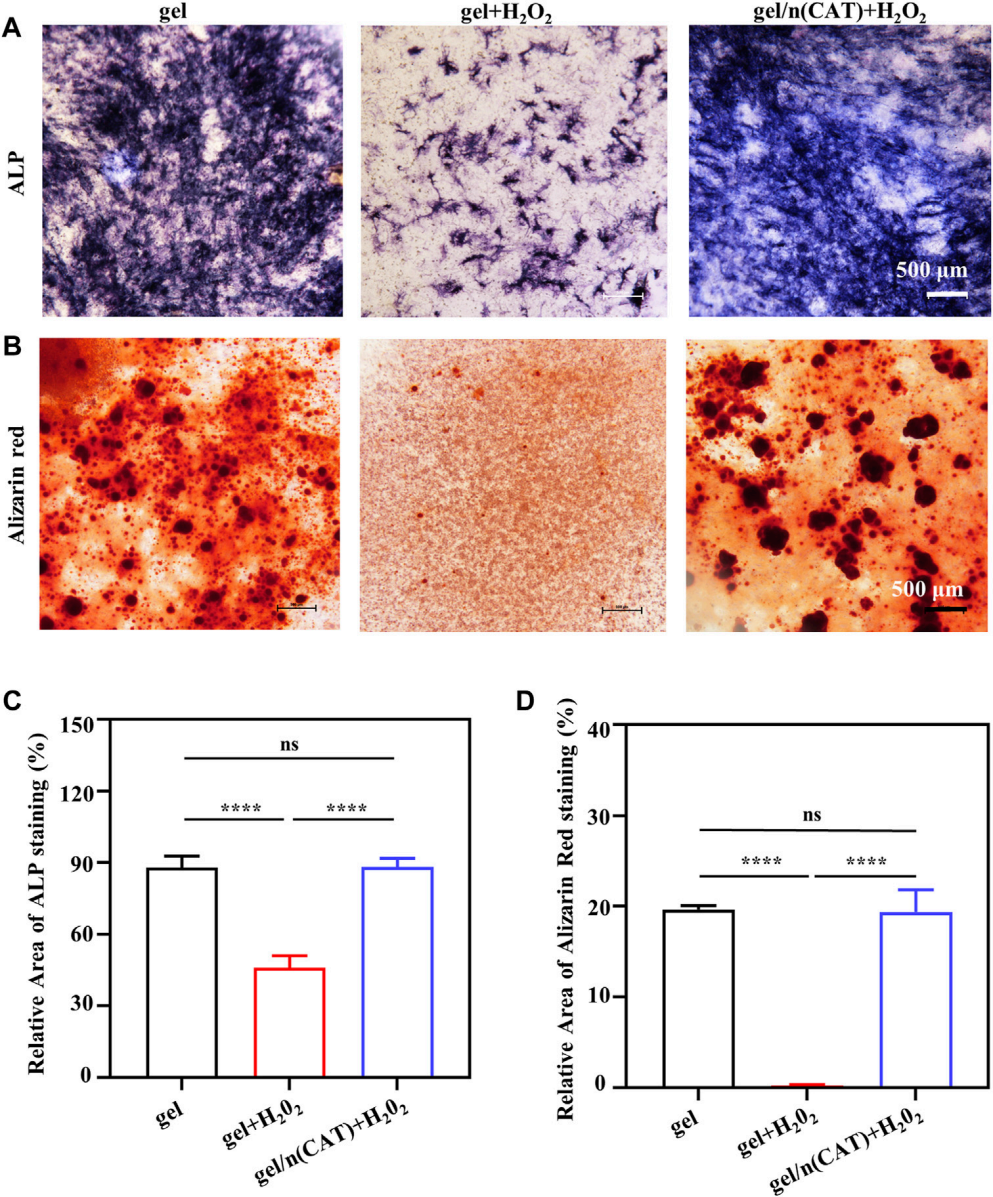
Osteogenic differentiation of osteoblasts by gel-n (CAT) in a persistent chronic inflammatory environment *in vitro*. **(A)** ALP staining of induced BMSCs for 7 days. **(B)** Alizarin Red Staining of induced BMSCs for 14 days. **(C)** Quantitative analysis of ALP and **(D)** Alizarin Red Staining images were analyzed by ImageJ software. (n = 3, ns means that there are no significant differences, ****p < 0.0001).

Figure 4A shows the ALP staining of osteoblasts on the gel surface under different environments. The degree and extent of ALP staining are significantly reduced in the chronic inflammatory environment compared to the gel in the normal environment. This is due to that the persistent chronic inflammatory environment greatly inhibits osteogenic differentiation of osteoblasts. While ALP staining of osteoblasts on the surface of gel/n (CAT) in the same persistent chronic inflammatory environment showed a significant increase in the degree and extent of ALP staining compared to gel **(** Figure 4A), indicating that n (CAT) effectively relieved inhibition of osteogenic differentiation of osteoblasts by the chronic inflammatory environment. The ARS staining results showed the same trend as the ALP staining (Figure 4B). Furthermore, by quantifying the area of ALP and ARS staining (Figures 4C, D), it was found that the area percentage of ALP staining and ARS staining of cells on the gel surface in the persistent chronic inflammatory environment was reduced to 45.97 and 0.25 compared to 88.31 and 19.63 in the normal environment; while for osteoblasts on the gel/n (CAT) surface in the same chronic inflammatory environment, the staining area of ALP and ARS up to 88.21 and 19.35 were not significantly different from the area of ALP and ARS staining of osteoblasts on the gel surface in the normal environment (Figures 4C, D). All the results suggest that gel/n (CAT) has an excellent ability to contribute to bone differentiation in a chronic inflammatory environment.

### Effect of Ti-gel/n (CAT) on osteopontin expression in osteoblasts in a persistent chronic inflammatory environment *in Vitro*

**FIGURE 5:**
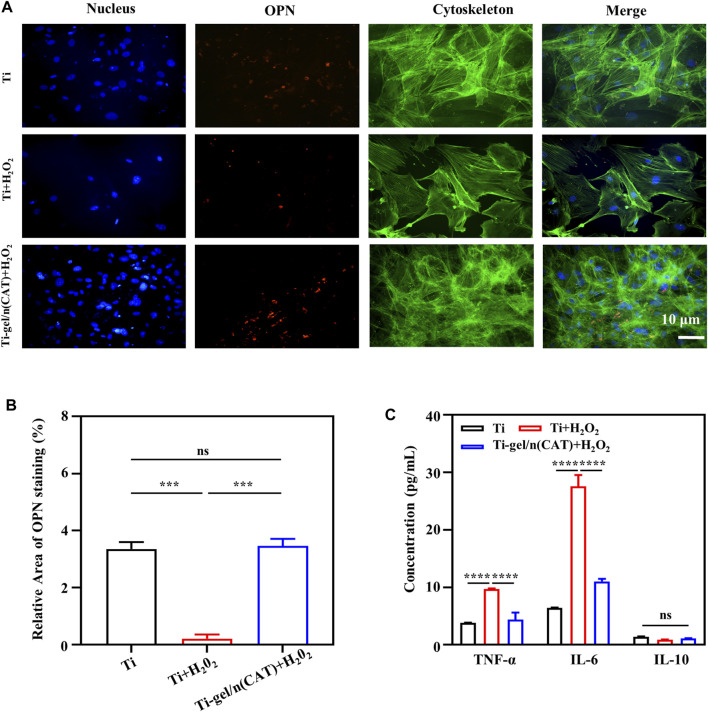
OPN expression by osteoblasts on the surface of different materials in a persistent chronic inflammatory environment. **(A,B)** Immunofluorescence images of OPN staining on different surfaces of the material. The OPN was stained in red, and the quantitative analysis of the OPN immunofluorescence images was analyzed using ImageJ software. **(C)** Cytokine expression levels of macrophages on different material surfaces. (n = 3, ns means that there are no significant differences, ***p < 0.001, ****p < 0.0001).

Osteopontin (OPN) is closely associated with bone formation and development. To investigate the level of osteoblast OPN expression on the surface of different materials under persistent chronic inflammatory conditions. On day 7 of the osteogenic differentiation culture in a persistent chronic inflammatory environment, immunofluorescence staining was used to investigate the expression of the OPN protein by osteoblasts on the surface of the material in different environments. The intensity of OPN fluorescence of the Ti surface was significantly weaker than that under a normal environment **(** Figure 5A). On the intensity of the OPN fluorescence of the Ti-gel/n (CAT) surface under the same persistent chronic inflammatory environment was significantly enhanced compared to that of the Ti-gel (Figure 5B). Further quantification result of the percentage of OPN fluorescence area showed that the persistent chronic inflammatory environment led to a decrease in the percentage of OPN fluorescence area on the Ti surface from 3.35 to 0.204. While the percentage of OPN fluorescence area on the Ti gel/n (CAT) surface reached as high as 3.46, which was not significantly different from the percentage of OPN fluorescence area on the Ti surface in normal environment. The above results fully demonstrate that Ti-gel/n (CAT) can effectively increase OPN expression in a persistent chronic inflammatory environment **(** Figures 5A, B) thereby promoting osteoblast osteogenesis differentiation (Figures 4A, B).

In a persistent chronic inflammatory environment, H_2_O_2_ can act as a signaling molecule to trigger the release of a variety of cytokines and chemokines from immune-responsive cells to enhance the inflammatory response. At the same time, pro-inflammatory factor tumor necrosis factor-α (TNF-α), pro-inflammatory factor interleukin-6 (IL-6), and anti-inflammatory factor interleukin-10 (IL-10), which are secreted by monocytes such as macrophages, play an important role in the inflammatory response (Qin et al., 2020; Li et al., 2021; Yan et al., 2022). To further explore the effect of Ti-gel/n (CAT) on the expression levels of inflammatory cytokines associated with macrophages RAW267.4 in a chronic inflammatory setting. As shown in Figure 5C, the expression levels of TNF-α and IL-6 both increased significantly in macrophages on the Ti surface in a persistent chronic inflammatory environment compared to the Ti surface in a normal environment, indicating that a persistent chronic inflammatory environment improved the expression of relevant pro-inflammatory factors. In the same chronic inflammatory environment, the expression levels of TNF-α and IL-6 cytokines were significantly reduced in macrophages on the surface of Ti-gel/n (CAT) compared to Ti. These results demonstrate that Ti-gel/n (CAT) can effectively reduce the expression of pro-inflammatory factors in the persistent chronic inflammatory environment, and its mechanism of contributing to bone in the persistent chronic inflammatory environment *in vivo* is likely to be achieved by reducing the expression levels of pro-inflammatory factors.

## Conclusion

In summary, we have developed a strategy for surface functionalization of titanium alloys through functional coating of the Ti surface with chitosan gel containing nAg and n (CAT), in which nAg significantly enhanced the antibacterial ability of the Ti surface, and n (CAT) effectively promoted osteogenic differentiation of osteoblasts in a persistent chronic inflammatory environment. In addition, as a general approach, our strategy can be extended to the bioactive functionalization of other implants for various medical applications.

## Data Availability

The original contributions presented in the study are included in the article/Supplementary Material, further inquiries can be directed to the corresponding authors.
