# Supplementary material for: Functional chitosan gel coating enhances antimicrobial properties and osteogenesis of titanium alloy under persistent chronic inflammation
Source: Front Bioeng Biotechnol. 2023 Feb 15;11:1118487. doi: 10.3389/fbioe.2023.1118487 (PMC9976779; doi:10.3389/fbioe.2023.1118487)
Supplement: Supplementary file 1 [file DataSheet1.docx]

Supplementary Material

**Functional chitosan gel coating enhances antimicrobial properties and osteogenesis of titanium alloy under persistent chronic inflammation**

Ti Zhang^1,2†^, Xiaoyan Qin^3†^, Yuan Gao^4†^, Dan Kong^4^, Yuheng Jiang^1,6^, Xiang Cui^1,2^, Miantong Guo^3^, Junyu Chen^1,2^, Feifan Chang^1,2^, Ming Zhang^5*^, Jia Li^1,2*^, Pengbin Yin^1,2^

*** Correspondence:**

Corresponding Author:

Jia Li, leejia301@126.com

Ming Zhang, [zhangming1@pkuih.edu.cn](mailto:zhangming1@pkuih.edu.cn)


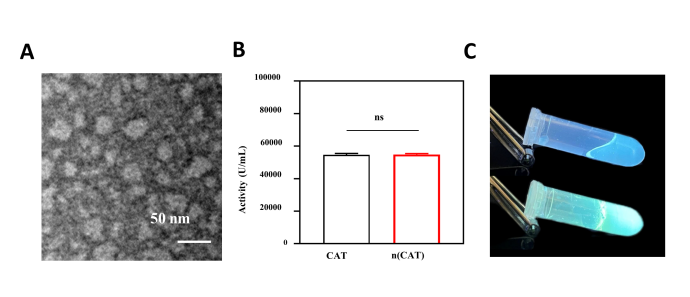


**Supplementary Figure 1.** (A) TEM image of n(CAT), (B) The enzyme activity of CAT and n(CAT), (C) Physical image of gel/nAg+n(CAT).


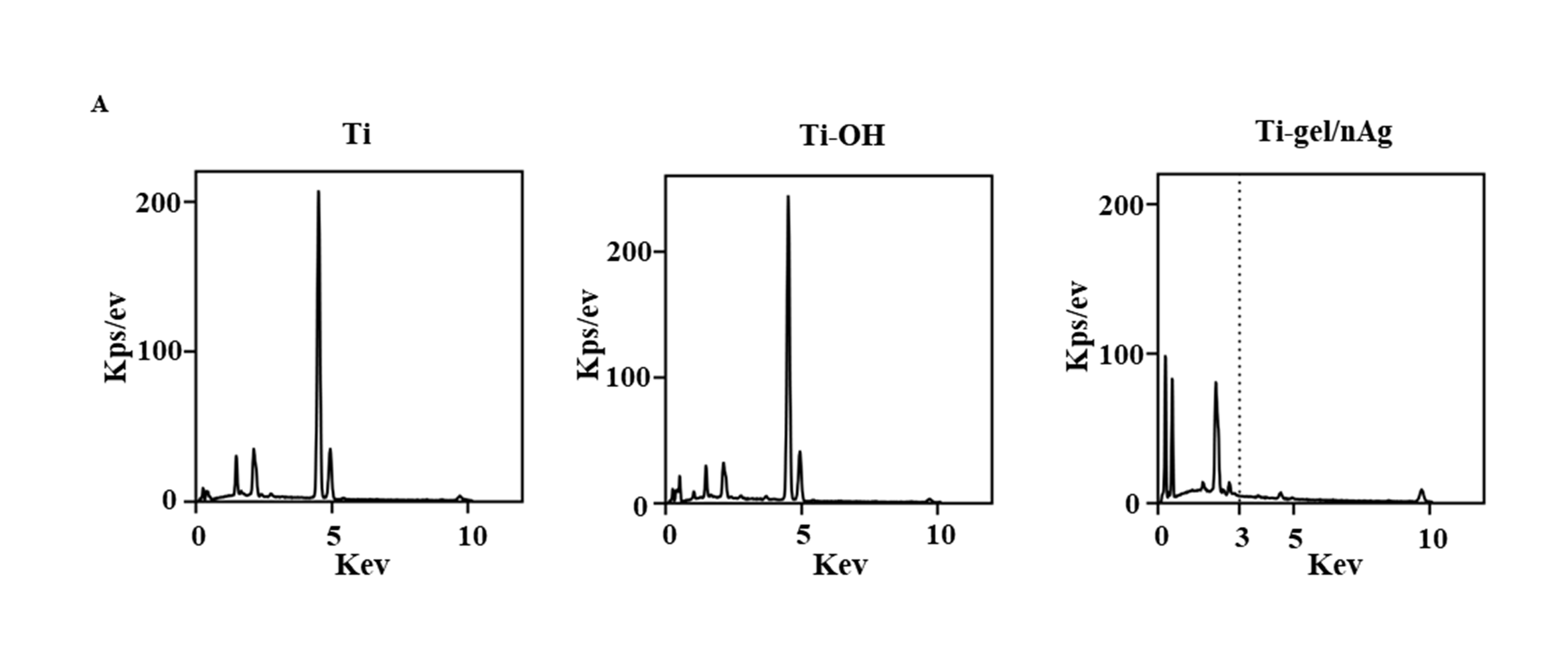


**Supplementary Figure 2.** (A) The EDS of Ti, Ti-OH and Ti-gel/nAg.
